# Experimental validation of a portable tidal volume indicator for bag valve mask ventilation

**DOI:** 10.1186/s42490-022-00066-y

**Published:** 2022-11-17

**Authors:** Benjamin S. Maxey, Luke A. White, Giovanni F. Solitro, Steven A. Conrad, J. Steven Alexander

**Affiliations:** 1 Department of Molecular & Cellular Physiology, LSU Health Shreveport, 1501 Kings Highway, Shreveport, LA 71103-3932 USA; 2 Department of Orthopaedic Surgery, LSU Health Shreveport, Shreveport, LA USA; 3 Department of Medicine, LSU Health Shreveport, Shreveport, LA USA; 4 Department of Emergency Medicine, LSU Health Shreveport, Shreveport, LA USA; 5 Department of Pediatrics, LSU Health Shreveport, Shreveport, LA USA; 6 Department of Neurology, LSU Health Shreveport, Shreveport, LA USA

**Keywords:** Ventilation, Emergency, Low-cost, Lung, Tidal volume, Monitor, Bag valve mask, Manual ventilation

## Abstract

**Introduction:**

Short-term emergency ventilation is most typically accomplished through bag valve mask (BVM) techniques. BVMs like the AMBU^®^ bag are cost-effective and highly portable but are also highly prone to user error, especially in high-stress emergent situations. Inaccurate and inappropriate ventilation has the potential to inflict great injury to patients through hyper- and hypoventilation. Here, we present the BVM Emergency Narration-Guided Instrument (*BENGI*) – a tidal volume feedback monitoring device that provides instantaneous visual and audio feedback on delivered tidal volumes, respiratory rates, and inspiratory/expiratory times. Providing feedback on the depth and regularity of respirations enables providers to deliver more consistent and accurate tidal volumes and rates. We describe the design, assembly, and validation of the BENGI as a practical tool to reduce manual ventilation-induced lung injury.

**Methods:**

The prototype BENGI was assembled with custom 3D-printed housing and commercially available electronic components. A mass flow sensor in the central channel of the device measures air flow, which is used to calculate tidal volume. Tidal volumes are displayed via an LED ring affixed to the top of the BENGI. Additional feedback is provided through a speaker in the device. Central processing is accomplished through an Arduino microcontroller. Validation of the BENGI was accomplished using benchtop simulation with a clinical ventilator, BVM, and manikin test lung. Known respiratory quantities were delivered by the ventilator which were then compared to measurements from the BENGI to validate the accuracy of flow measurements, tidal volume calculations, and audio cue triggers.

**Results:**

BENGI tidal volume measurements were found to lie within 4% of true delivered tidal volume values (95% CI of 0.53 to 3.7%) when breaths were delivered with 1-s inspiratory times, with similar performance for breaths delivered with 0.5-s inspiratory times (95% CI of 1.1 to 6.7%) and 2-s inspiratory times (95% CI of –1.1 to 2.3%). Audio cues “*Bag faster*” (1.84 to 2.03 s), “*Bag slower*” (0.35 to 0.41 s), and “*Leak detected*” (43 to 50%) were triggered close to target trigger values (2.00 s, 0.50 s, and 50%, respectively) across varying tidal volumes.

**Conclusions:**

The BENGI achieved its proposed goals of accurately measuring and reporting tidal volumes delivered through BVM systems, providing immediate feedback on the quality of respiratory performance through audio and visual cues. The BENGI has the potential to reduce manual ventilation-induced lung injury and improve patient outcomes by providing accurate feedback on ventilatory parameters.

**Supplementary Information:**

The online version contains supplementary material available at 10.1186/s42490-022-00066-y.

## Introduction

Bag valve mask (BVM) ventilation remains one of the most widely used techniques for providing short-term and emergency manual ventilation both in hospital and pre-hospital settings [1]. Of particular importance is the BVM’s potential utility in austere medical environments where advanced respiratory support is limited by availability of medical equipment, supplies, and infrastructure [2–4]. Medical accessibility issues have been highlighted by supply chain disruption during the ongoing COVID-19 pandemic [5]. Recent research efforts have been directed at automation of BVM ventilation to improve their safety and efficacy, and expand their utility to alleviate resource shortages [6–8].

Although simple in concept, effective ventilation with BVM systems still requires extensive training, and potential misuse can significantly diminish the BVM’s efficacy and safety [9, 10]. Hypoventilation and inadequate gas exchange are important concerns during manual ventilation, but hyperventilation remains a more common and dangerous user error that can lead to serious injury [11]. Delivering too great a tidal volume (the volume of air provided to the patient during one round of inspiration) increases the risk for potentially serious adverse effects, including direct volutrauma to the lungs leading to pneumothorax [8, 12, 13], gastric insufflation and aspiration [14, 15], and altered hemodynamics [16–19].

Reports show that lower volume ventilation can yield lung-protective effects in several clinical scenarios [20–24]. However, even ‘properly’ trained personnel tend to over-bag patients, meaning that tidal volumes are too high and/or the ventilation rate is too rapid. Without objective feedback, providers cannot reliably assess the quality of their respirations during manual ventilation. Studies estimate that in up to 80% of cases, healthcare providers (irrespective of their level of training) will over-ventilate patients [9–11, 25–27].

Several devices have been developed which provide some level of feedback to healthcare providers during manual ventilation, but are generally limited by high manufacturing/commercial costs, portability, difficulty in quick transition from one patient to another, and complex user interfaces which require extensive training and additional user interpretation [28–32].

Here, we propose a user-friendly, cost-effective tidal volume and respiratory rate feedback device – the BVM Emergency Narration-Guided Instrument (BENGI) – optimized for high-volume, resource-scarce emergent care. The aim of this study is to validate the functionality of the BENGI. For the BENGI to be considered an effective ventilation feedback device, it must accurately measure airflow, calculate tidal volumes, and clearly convey instructions to improve ventilation (alerting the user when a target tidal volume has been reached, when to begin and end inspirations to achieve a certain respiratory rate, and if a mask leak is detected).

## Materials and methods

### Design

#### Product requirements

Primary requirements for the BENGI include accurate measurement of air flow, continuous calculation of delivered tidal volumes, continuous display of cumulative delivered tidal volume during a given respiratory cycle and playing of audio cues that alert the user 1) when to begin and end inspiration, 2) when inspiratory time exceeds or does not meet safe thresholds, and 3) when an air leak has been detected in the respiratory circuit. After these requirements were satisfied, several secondary goals guided remaining design decisions.

We designed the BENGI to be radially symmetric so that attachment to the bag and mask could be achieved rapidly without need of adjustment to effectively view visual cues. Designing the flow measurement system to be impermanently attached to the BENGI minimizes cross-contamination between uses. When the BENGI is connected to a BVM system, the only portion of the device in contact with the respiratory circuit is the flow meter and accessory 3D-printed cylinder. The flow meter is also disposable, allowing all parts that are exposed to patients to be completely removed and disposed or cleaned between patient use. Patient safety, as well as ease in transitioning the device from one patient to another, motivated this design decision.

**Fig.1:**
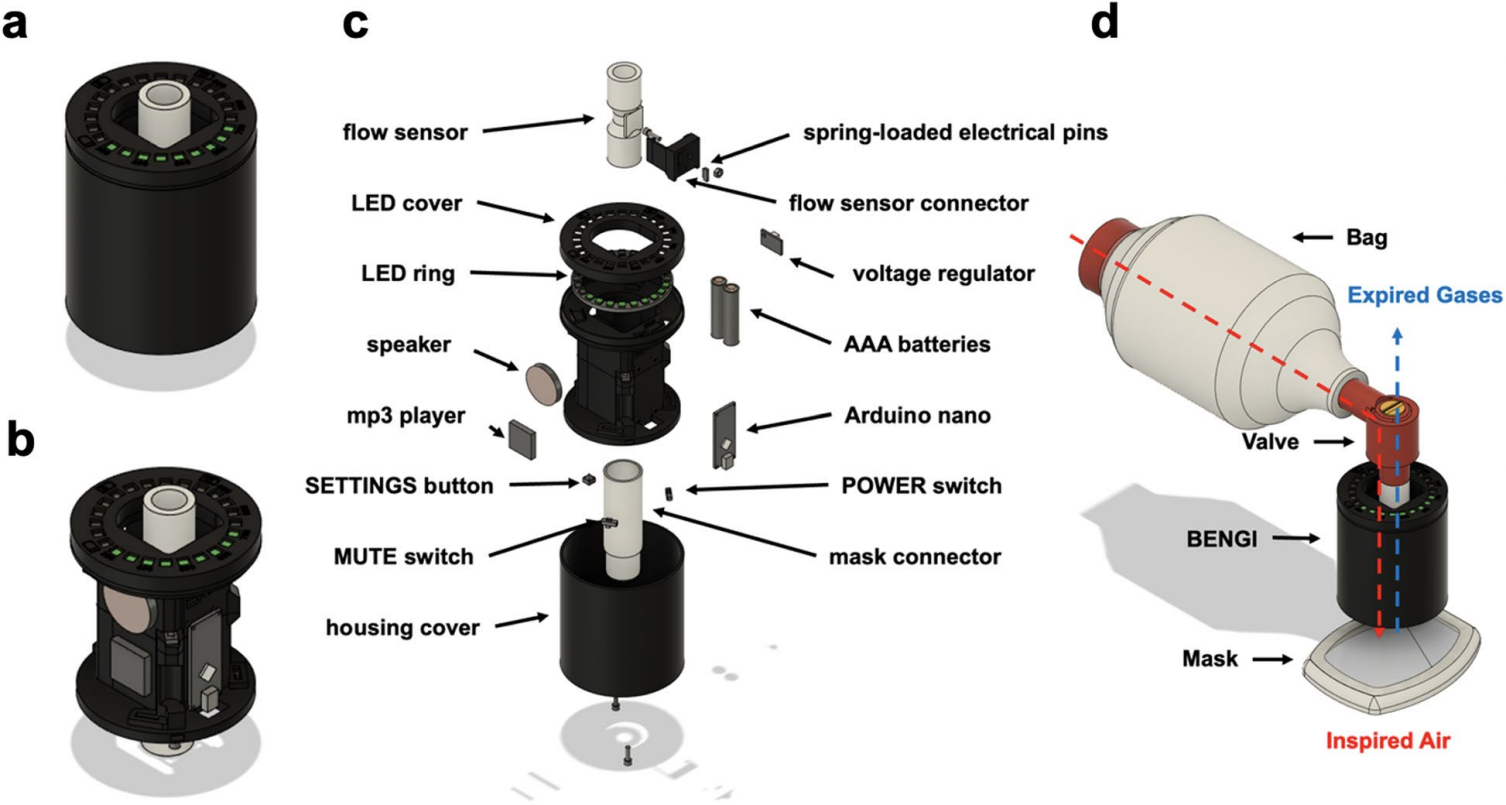
Computer aided design drawings of the assembled BENGI, both with **a** and without **b** the housing cover. **c** An exploded view of the BENGI with labeled internal components. **d** The incorporation of the BENGI into a bag valve mask system

As the BENGI is intended to be optimized for resource-scarce emergent care, low cost of assembly motivated specific part choices. At the time of writing, the BENGI prototype was assembled with commercially available electronic components for < 100 USD (Fig. 1).

#### User interface

Inputs to the system include: 1) a digital mass flow sensor (Sensirion; SFM3300-D), 2) a single pole single throw “MUTE” switch to silence audio, 3) a circuit that measures battery life, and 4) a “SETTINGS” push button that cycles through adjustments to user settings and displays battery life. Outputs include a speaker/MP3 player, as well as an LED pixel ring to display tidal volume data or current settings and battery life.

In the BENGI’s current design, the user can quickly and easily set a range of target tidal volumes (*V*_*T*_) between 300 and 900 mL in 50 mL increments via the SETTINGS button on the bottom surface of the device. When the user begins delivering a breath through the BENGI, pixels on the LED ring begin lighting up, progressing clockwise around the ring. The number and color of lit pixels at a given time corresponds to the fraction of the target *V*_*T*_ that the user has delivered. The use of LEDs versus a display screen for visual cues is simple, interpretable, and easily visible in a variety of conditions while simultaneously keeping unit costs lower compared to the use of display screens, allowing for a longer battery life.

**Fig. 2:**
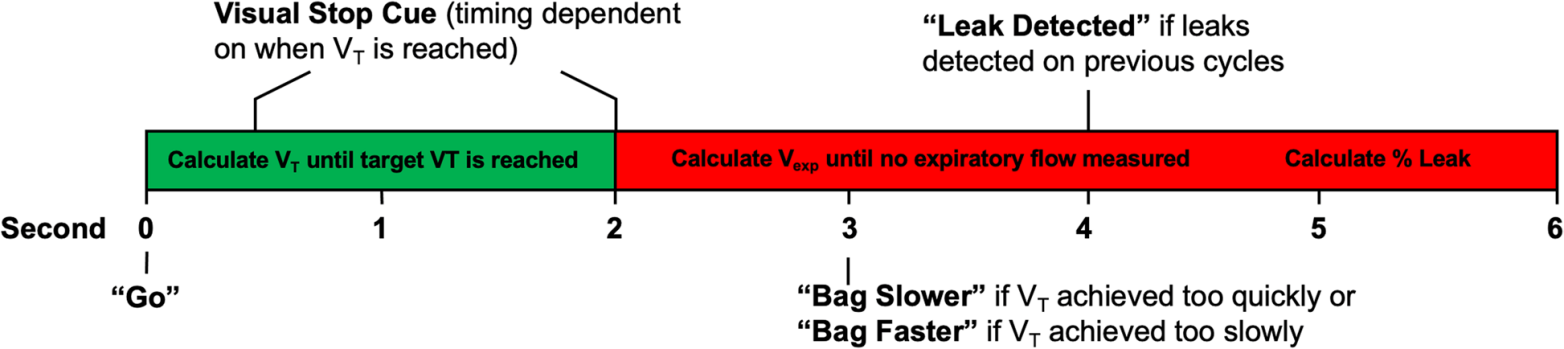
BENGI visual and audio cues (in quotations), timings, and triggers during the 6 s respiratory cycle. Time in inspiration and expiration represented with green and red rectangles with calculations performed in real-time (text inside boxes)

In addition to displaying delivered tidal volumes, the BENGI gives instruction and feedback on the rate and quality of respirations through audio cues. Every 6 s (10 cycles per minute), the BENGI plays an audio cue saying, “*Go*,” which instructs the user to begin an inspiration. The BENGI may play audio cues that instruct the user to “*Bag faster”* or “*Bag slower*” if the duration of the previous inspiratory cycle was longer than 2 s or faster than 0.5 s, respectively. The “*Bag slower*” cue will also play if the maximum flow value for the previous inspiration exceeded 60 L per minute. Finally, the BENGI may play an audio cue that indicates if an air leak is present in the system (*“Leak detected”*) if expiratory volume through the flow sensor is less than 50% of the previous inspiratory volume in three consecutive cycles (Fig. 2).

#### Working Logic

Central processing is performed by an Arduino Nano Every (ATMega4809 microcontroller), which was chosen primarily for its ease of use and low cost. The device’s software operates on a continuous loop. The loop is separated into three main sections. First, timers and counters are updated, based on results from the previous loop, and any cued audio is played. Data are then read from the mass flow sensor, which is used to determine the state of the BENGI (idle, inspiration, or expiration) and calculate tidal volumes delivered through the device. Finally, either the current delivered *V*_*T*_ or the device’s status (target *V*_*T*_ setting and battery life) are displayed on the LED ring, depending on whether the “SETTINGS” button has been pressed.

The BENGI functions in 3 distinct states: (1) idle, (2) inspiration, and (3) expiration. One respiratory cycle consists of moving consecutively from idle, to inspiration, to expiration, and finally back to the idle state. On each cycle through the main software loop, one flow measurement is read from the mass flow meter. The 25 most recent flow readings are stored to aid in determining whether the device is in an idle, inspiratory, or expiratory state. After updating the state, the device calculates either the tidal (if in inspiration) or expired (if in expiration) volume. Upon switching from idle to inspiration, the previous 25 flow measurements are retroactively summed using a non-recursive trapezoidal Riemann sum approximation. Throughout the rest of inspiration, a running total of tidal volume is calculated using a recursive trapezoidal Riemann sum approximation. When switching from inspiration to expiration and throughout expiration, identical recursive trapezoidal Riemann sum calculations are performed to calculate the expired volume.

Additionally, at the transition from expiration to idle, the device makes an adjustment to the displayed target tidal volume *V*_*T,display*_ to compensate for the user’s reaction time. Different users may react more quickly or slowly to the LED visual cues, and making slight adjustments to *V*_*T,display*_ may account for this variability. If the user is reacting slowly to the visual cues, then the delivered tidal volume may overshoot the target. To compensate, *V*_*T,display*_ is adjusted so that the visual cues are shown sooner. Conversely, if the user is reacting more quickly, the delivered tidal volume may undershoot the target, and *V*_*T,display*_ is altered so that visual cues are shown later.

#### Casing and power supply

The hardware layout and housing for the BENGI were designed using computer aided design software (Fusion 360 V.2.0.12376, Autodesk, San Rafael, CA, USA). Rapid design prototyping and revision for the BENGI housing was achieved using the in-house fused filament deposition (eSUN black 1.75 mm PLA + filament with the Prusa i3 MKS + , Prusa, Prague, Czech Republic) and stereolithography (surgical guide resin printed with the Form 2 printer, Formlabs, Somerville, MA, USA) 3D printing. PLA is a highly biocompatible material commonly used for medical device construction and withstands cleaning well [33]. The unit is powered by AAA batteries, and a battery health check feature has been implemented.

An SLA 3D printed rigid tube (surgical guide resin) connects the flow sensor and mask while only adding an additional 20 mL of “dead” airspace. Spring-loaded metal pins affixed inside the clip make electrical contact with the pads on the surface of the flow meter.

All electronic components in the BENGI require 5 V input, but the device is powered by 3 AAA batteries (only 4.5 V in series). Adequate voltage is generated through a 5 V booster (Texas Instruments; TPS61023), which takes inputs of 2 V to 5 V and outputs a steady 5.2 V. A toggle “POWER” switch turns the device on and off. The decision to use AAA batteries was motivated largely by cost and space requirements, as power sources at and above 5 V are generally more expensive and larger.

### Validation

Several benchtop tests were performed to validate the BENGI’s components and algorithms. Two potential causes for inaccurate tidal volume readings by the BENGI were identified: inaccurate flow measurements and inadequate sampling rate of the flow measurement. Each were tested independently. After, direct testing of the BENGI’s tidal volume measurement accuracy was performed. Finally, the accuracy of the audio cue triggering was tested.

#### BENGI flow measurement validation

An oxygen tank equipped with a two-stage pressure regulator was connected to the inlet sides of the flow sensor for the BENGI and computer-connected sensor arranged in series with silicone rubber fitted CPAP hosing (6 ft. × 19 mm inner diameter, Philips Respironics, Murrysville, PA) and conventional corrugated ventilator tubing. The computer-connected sensor was a second Sensirion SFM3300-D flow meter calibrated and operated through software developed by the manufacturer. Thus, comparisons in measurements between the BENGI’s sensor and the computer-connected sensor reflect potential differences in calibration and data processing. The outlet side of the computer sensor was vented to atmosphere, allowing for unobstructed flow. The outlet pressure on the regulator was adjusted to achieve varying flow rates from approximately 0 to 90 standard liters per minute (slm) in 10 slm increments, as measured by the computer sensor. Standard liters per minute are defined for a gas at standard temperature and pressure (STP: 0º C, 100 kPa). The sensor assumes gases are at STP. However, since both the computer and BENGI sensors are simultaneously measuring the same gas source, variations in temperature and pressure from STP would affect each sensor’s output the same (assuming negligible changes in temperature and pressure between the sensors), allowing for a valid comparison. The measured flow was recorded for 100 ms with both the BENGI (via serial communication with the computer) and with a standalone sensor run on the manufacturer’s software, and the flow measurements were then compared.

#### BENGI flow sampling rate validation

To determine the flow sampling rate achieved by the BENGI, the BENGI was connected in-line between an adult BVM (SPUR II^®^ adult model, AMBU^®^ A/S, Columbia, MD, USA) and a calibrated test lung (Calibrated QuickLung^®^, IngMar Medical Pittsburg, PA, USA) with silicone rubber fitted CPAP hosing and conventional corrugated ventilator tubing. Breaths were then delivered to the test lung by manually squeezing the BVM, following the visual and audio cues presented to the user by the BENGI (target *V*_*T*_ = 500 mL). The number of times that the main software loop was completed during 1-s intervals were recorded internally on the BENGI and sent to the computer via USB transmission during bagging. Since the flow is measured once per loop, the number of times the main software loop was traversed correlates one to one with the number of flow measurements made.

To estimate the effect that altering sampling rate would have on tidal volume measurement accuracy, the computer flow sensor was connected in-line between the adult BVM and a test lung with silicone rubber fitted CPAP hosing and conventional corrugated ventilator tubing. A single breath was delivered via the BVM at a tidal volume of approximately 500 mL over an inspiratory time of approximately 1 s, and the flow measurements (*n* = 2151 samples) of the inspiratory portion of the respiratory cycle were recorded with the computer sensor. This data set was then artificially down-sampled with varying sample numbers. These down-sampled data were used to calculate new tidal volumes, which was then compared to the measured tidal volume.

#### BENGI tidal volume measurement validation

To determine the BENGI’s accuracy at measuring *V*_*T*_, the BENGI and computer flow sensor were connected in-line in series between a mechanical ventilator (Engström Carestation™, General Electric Healthcare, Chicago, IL) and the test lung. The ventilator was set in mandatory volume control ventilation, and breaths were delivered to the test lung at a constant respiratory rate (10 breaths/min) and varying inspiratory times (*t*_*insp*_ = 0.5, 1.0, and 2.0 s) and tidal volumes (*V*_*T*_ = 300 to 900 mL in 50 mL increments). Flow measurements from at least 10 consecutive respiratory cycles were captured with the computer flow sensor. The flow waveform was analyzed with custom code written in Python (version 3.7.2, Python Software Foundation, Beaverton, Oregon, United States) to determine inspiratory start and end times and calculate *V*_*T*_. The onboard, real-time calculation of *V*_*T*_ by the BENGI was sent to the computer at end inspiratory time via USB transmission. The computer and BENGI calculated *V*_*T*_s were matched based upon end inspiration times, and the measurement differences in *V*_*T*_ were analyzed by Bland–Altman plot analysis.

#### BENGI audio cue triggering validation

The audio cues “*Bag slower*” and “*Bag faster*” were to be triggered when the measured *t*_*insp*_ is too slow (*t*_*insp*_ > 2.0 s) or too fast (*t*_*insp*_ < 0.5 s), respectively. To validate that the audio cues appropriately trigger, the BENGI was again connected in-line to the test lung and mechanical ventilator. The ventilator was set in mandatory volume control ventilation (respiratory rate = 5 breaths/min) at varying tidal volumes (*V*_*T*_ = 300, 500, and 750 mL). To determine the *t*_*insp*_ that the “*Bag slower*” audio cue was triggered for a given *V*_*T*_, *t*_*insp*_ was set to 0.5 s and allowed to cycle through at least 10 respiratory cycles. If the cue was triggered at least once out of the 10 respiratory cycles, then *t*_*insp*_ was incrementally increased at the smallest increment achievable with the mechanical ventilator until the cue failed to trigger. Then, *t*_*insp*_ was incrementally decreased until the audio cue was triggered for all 10 respiratory cycles. This *t*_*insp*_ was defined as the lower bound time for triggering the “*Bag slower*” audio cue. The upper bound time — the fastest *t*_*insp*_ for which the “*Bag faster*” audio cue was not triggered in at least one respiratory cycle — was then the previous *t*_*insp*_ increment. The average *t*_*insp*_ that triggers the “*Bag slower*” cue must then lie between the lower and upper bound times. This process was repeated for the “*Bag faster*” audio cue with an initial *t*_*insp*_ set to 2.0 s. Cue triggers were reported via serial communication from the BENGI.

To test the sensitivity of the BENGI’s leak detection algorithm, the ventilator was set in mandatory volume control ventilation (respiratory rate = 10 breaths/min, *t*_*insp*_ = 1.0 s) at varying tidal volumes (*V*_*T*_ = 300, 500, and 750 mL). Holes of 1.5 mm diameter were sequentially drilled into the corrugated ventilator tubing between the expiratory side of the BENGI’s flow sensor and the test lung to cause increasing levels of leak, which was detected and calculated as a percent of *V*_*T*_ by the mechanical ventilator. If a leak was detected by the BENGI, then a signal was transmitted to the computer from the BENGI via USB. The upper limit was defined as the percent leak for which the “*Leak detected*” signal was triggered for 10 out of 10 respiratory cycles. The lower limit was then defined as the percent leak achieved at the previous number of drilled holes, where at least 1 out of the 10 respiratory cycles did not trigger a “*Leak detected*” signal.

#### Statistics

Data are presented as mean ± standard deviation, unless otherwise noted. Pearson r correlation coefficients, log–log data transformation, simple linear regression analysis, D’Agostino and Pearson test for normality and Bland–Altman analysis were performed with the built-in functions from Graphpad prism statistics software (version 9.3, Graphpad, San Diego, CA).

## Results

### Device assembly and initialization

**Fig. 3:**
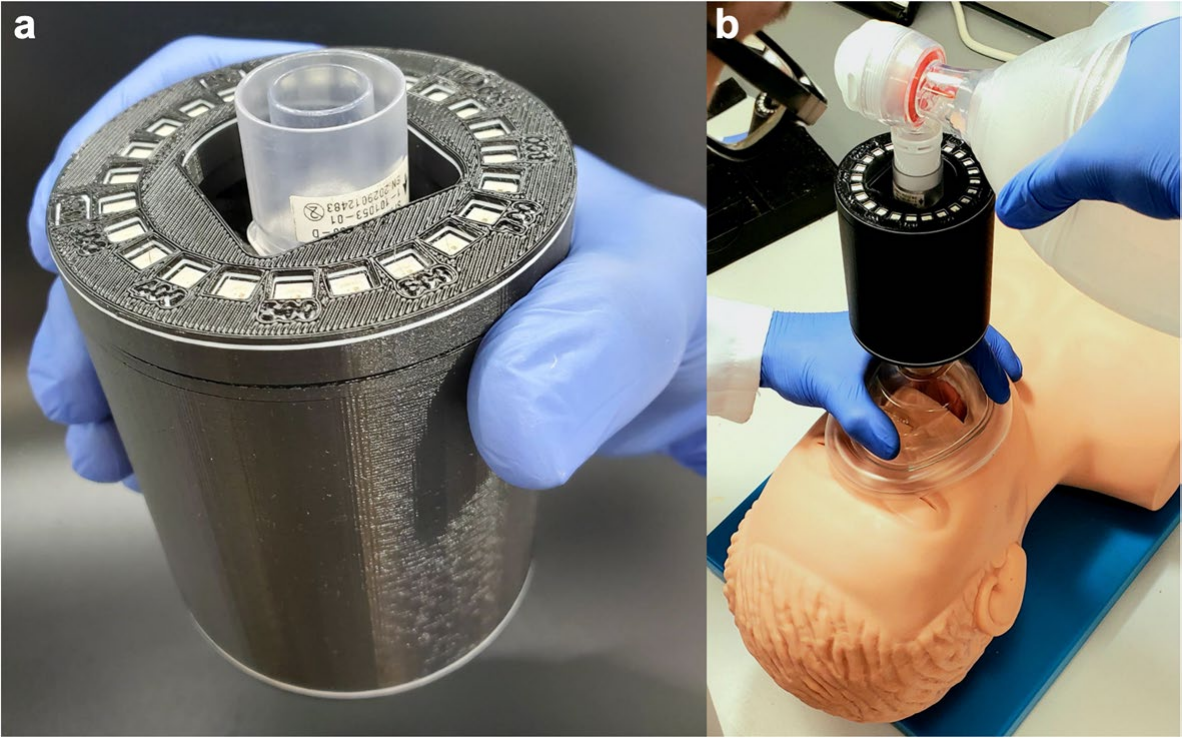
**a** Assembled BENGI prototype. **b** Demonstration of the BENGI with a bag valve mask (BVM) and manikin

The BENGI was successfully constructed from the 3D printed housing and electronic parts as designed (Fig. 3a). With the outer cover removed, a flow sensor could be installed into the flow sensor connector by hand and removed with relative ease. The 3D printed extension tube could be tightly connected to the flow sensor, allowing for attachment of the BENGI between a BVM bag and mask (Fig. 3b).

**Fig. 4:**
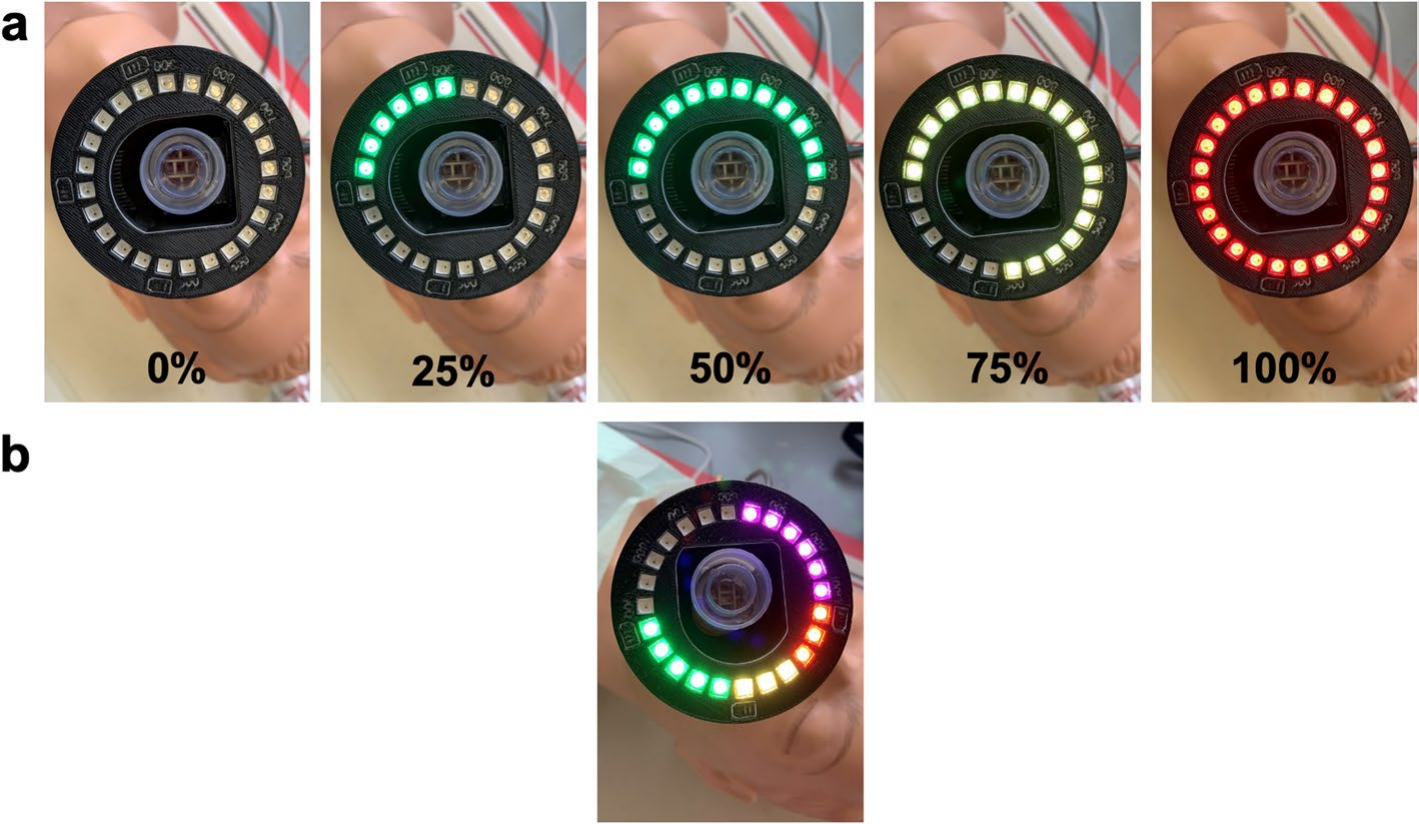
**a** Visual cues produced by the LED ring at varying levels of target *V*_*T*_ achieved (shown as % *V*_*T*_). As an inspiration is delivered, the LEDs light up consecutively around the ring, changing from green (between 0 to 50% target *V*_*T*_), to yellow (50% to 100%), and finally to red (> 100%) signaling that the full target tidal volume has been delivered. *V*_*T*_ = tidal volume. **b** LED display during status mode. The top half of the LED ring displays the current tidal volume setting via purple lights aligned with volumes (in mL) printed into the casing of the BENGI. The bottom half of the LED ring displays battery life, with green, yellow, and red together representing full battery, yellow and red together representing medium battery life, and red alone representing low battery

Flipping the POWER switch turned on the unit and the program initialized as expected, with the LEDs briefly lighting up blue to indicate power. The LED ring produced signals appropriately during *V*_*T*_ delivery, subsequently lighting up and changing from green to yellow and finally to red (Fig. 4a). Depressing the SETTINGS push button switched the device to display mode, where the LED lit up to display the current target *V*_*T*_, as a partial ring of purple LEDs lit up to the current *V*_*T*_ label arranged around the periphery of the LED ring, and current battery power, as an opposing partial ring of red/yellow/green LEDs (Fig. 4b). Additionally, the audio cues could be muted and turned back on with the MUTE switch.

### Flow rate measurements

**Fig. 5:**
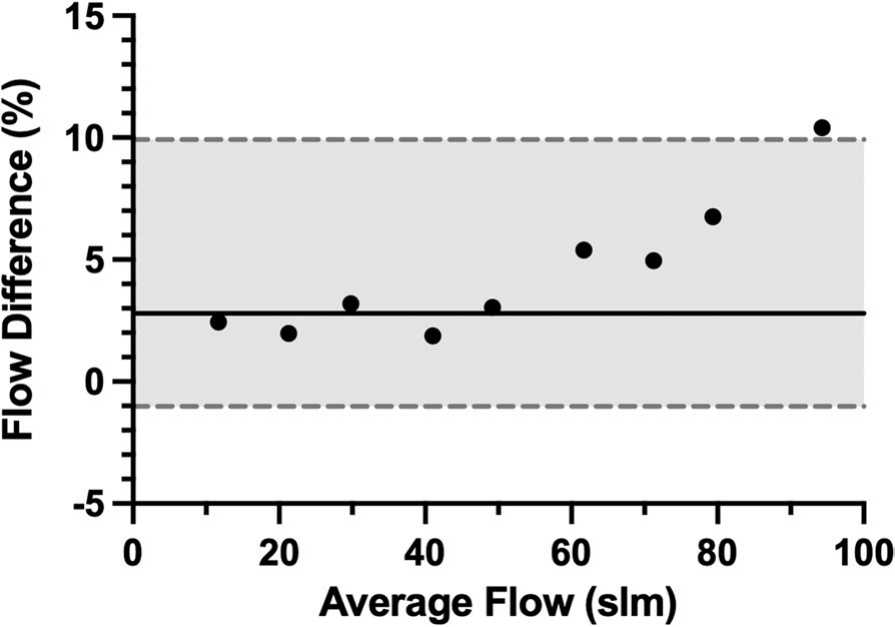
Bland–Altman plot of measured flows between the BENGI and computer (solid line indicates bias measurement; dotted line with gray shading represents 95% limits of agreement; Flow Difference calculated as Flow Difference = (BENGI flow – computer flow) / average flow)

Flow readings demonstrated a high correlation between the BENGI and computer sensor (Pearson *r* > 0.99). Percent differences between the flow measurements were normally distributed (*p* = 0.09), and subsequent Bland–Altman analysis (Fig. 5) showed that the BENGI had a bias (4.5%) to overestimate flow (95% limits of agreement from –1.0 to 9.9%). Furthermore, there is a clear trend for the BENGI to overestimate flow at higher flow rates, demonstrating a proportionality bias, such that the maximal difference in measured flow rate was 10.4% at 89 slm.

### Sampling rate simulation

**Fig. 6:**
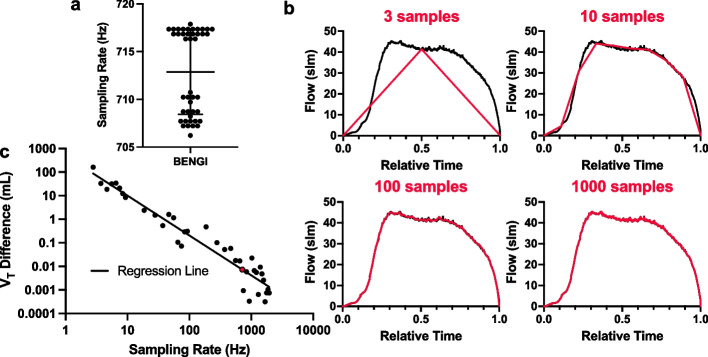
**a** Flow sampling rate of the BENGI measured during multiple (*n* = 43) 1-s intervals. Error bars indicate mean ± standard deviation. **b** Flow measurement profile captured by the computer sensor (black line) of a single inspiratory cycle from a BVM delivered to a test lung (measured t_insp_ = 1.08 s and *V*_*T*_ = 506 mL), and simulated flow measurement profiles captured at lower sampling rates (shown are sampling rates at 3, 10, 100, and 1000 samples of the full inspiratory time). **c** Log–Log plot of difference in calculated *V*_*T*_ from the simulated measurement profiles (3 to 10 samples at 1-sample intervals, 10 to 100 samples at 10 sample intervals, and 100 to 2100 samples at 100 sample intervals) to *V*_*T*_ calculated from the computer sensor versus the sampling rate (solid line indicates best-fit linear regression, *R*^2^ = 0.92). Red point indicates the predicted error between the BENGI *V*_*T*_ and computer, given the measured mean sample rate for the BENGI (716 Hz) and assuming flow measurements were in exact agreement between the BENGI and computer sensor for all time points

The flow sampling rate of the BENGI during 1-s intervals (*n* = 43) was measured, and the average flow sampling rate was 713 ± 4 Hz (Fig. 6a). The differences from the simulated *V*_*T*_*s* and the measured *V*_*T*_ at the maximum sampling rate (2 kHz, assumed to be the most accurate measurement of *V*_*T*_) appeared to decrease linearly on a log–log scale with increasing flow sampling rate (Fig. 6c, Pearson *r* = –0.96). Extrapolation of this relationship was then used to predict the error of the BENGI due to its lower flow sampling rate of 713 Hz, which was estimated to be 7 μL (Fig. 6c, red point).

### Tidal volume measurements

**Fig. 7:**
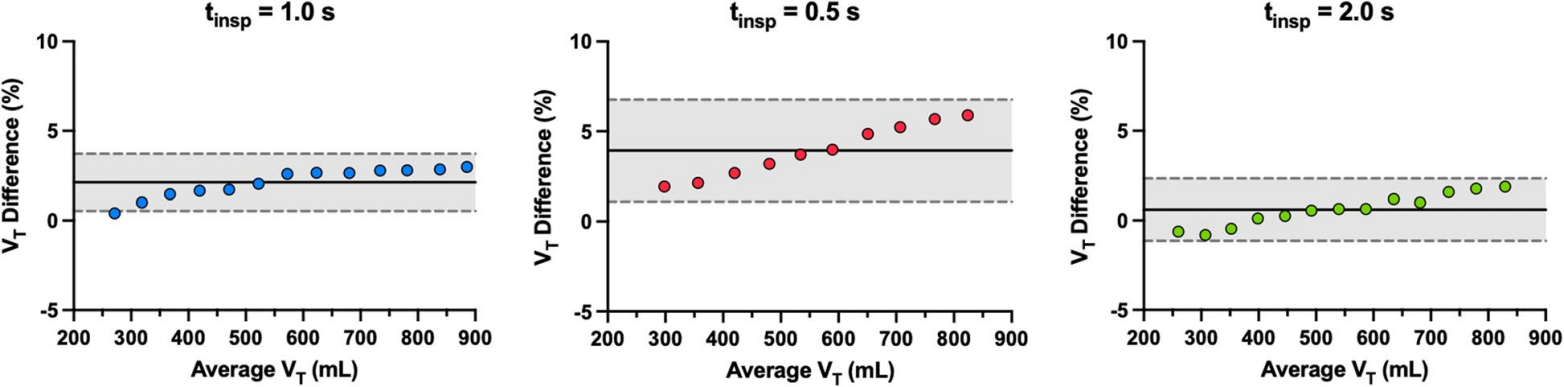
Bland-Altmann plots stratified by inspiratory time (1.0, 0.5, and 2.0 s, respectively; solid line indicates bias measurement; dotted lines with gray shading indicate the 95% limits of agreement; V_T_ Difference calculated as V_T_ Difference = (BENGI *V*_*T*_ – computer *V*_*T*_) / average *V*_*T*_)

The percent differences between the *V*_*T*_ calculated by the BENGI compared to the *V*_*T*_ computed from the computer flow sensor were normally distributed (*p* = 0.33, 0.38, and 0.64 for *t*_*insp*_ = 1.0, 0.5, and 2.0 s, respectively), and subsequent Bland–Altman analysis (Fig. 7) demonstrated a bias of 2.1% (95% limits of agreement = 0.53 to 3.7%), 3.9% (1.1 to 6.7%), and 0.6% (–1.1 to 2.3%), for *t*_*insp*_ = 1.0, 0.5, and 2.0 s, respectively. There is also a clear positive proportionality bias at all three *t*_*insp*_ that increases with larger *V*_*T*_s. At *t*_*insp*_ = 0.5 s, set *V*_*T*_s > 750 mL could not be achieved by the mechanical ventilator because the maximum pressure allowed by the ventilator (100 cm H_2_O) was exceeded.

### Audio cue triggers

The upper and lower bounds for the *t*_*insp*_ that triggered the “*Bag faster*” and “*Bag slower*” audio cues are shown in Table 1. The “*Bag slower*” audio cue was triggered at *t*_*insp*_ slightly faster than the set trigger time value of 0.5 s at *V*_*T*_ = 300 (between 0.35 to 0.41 s) and 500 mL (0.35 to 0.40 s). This means that *t*_*insp*_ > 0.5 s will not trigger the “Bag slower” audio cue at these *V*_*T*_s. Additionally, there may be some *t*_*insp*_ between the upper bound times and 0.5 s that will not trigger the “*Bag slower*” signal and remain undetected. The trigger for the “*Bag slower*” audio cue could not be tested at *V*_*T*_ = 750 mL because the maximum pressure allowed by the mechanical ventilator was exceeded for *t*_*insp*_ ≤ 0.5 s.

**Table 1:** Upper and lower bounds for inspiratory times (*t*_*insp*_) and percentages of tidal volumes (*V*_*T*_) that trigger “*bag slower/faster*” and “*leak detected*” audio cues, respectively, at *V*_*T*_ = 300, 500, and 700 mL. Upper and lower bounds represent intervals within which true values lie. The trigger for the “*Bag slower*” audio cue could not be tested at *V*_*T*_ = 750 mL because the maximum pressure allowed by the mechanical ventilator was exceeded for *t*_*insp*_ ≤ 0.5 s

**V**_**T**_	**“Bag slower”**		**“Bag faster”**		**“Leak detected”**	
	**Lower Bound (s)**	**Upper Bound (s)**	**Lower Bound (s)**	**Upper Bound (s)**	**Lower Bound (%V**_**T**_**)**	**Upper Bound (%V**_**T**_**)**
300	0.35	0.41	1.84	2.03	43	49
500	0.35	0.40	1.94	2.03	44	49
750	-	-	1.94	2.00	44	50

The “*Bag faster*” audio cue was triggered at *t*_insp_ between 1.84 to 2.03 s, 1.94 to 2.03 s, and 1.94 and 2.00 s for *V*_*T*_ = 300, 500, and 750 mL, respectively. Thus, *t*_insp_ greater than the upper bounds, which is ≤ 30 ms from the set trigger time of 2.0 s, will properly trigger the “*Bag faster*” audio cue at these *V*_*T*_s. Furthermore, there may be some *t*_*insp*_ between the lower bound times and 2.0 s that will inappropriately trigger the “*Bag slower*” signal.

### Leak detection

The upper and lower bounds of percent leak that trigger the “*Leak detected*” signal at varying *V*_*T*_s is also shown in Table 1 (between 43 to 49%, 44 to 49%, and 44 to 50% for *V*_*T*_ = 300, 500, and 750 mL, respectively) (Table 1). Thus, for all leaks greater than the set leak value = 50%, the “*Leak detected*” signal is triggered at these *V*_*T*_s. However, there may be some leaks between the lower bounds and 50% that inappropriately trigger the signal.

## Discussion

Our study demonstrates that the BENGI accomplishes several major designs targets, including 1) accurately measuring tidal volumes and flow rates, 2) clearly displaying tidal volume information, and 3) detecting poor quality respirations and leaks with subsequent delivery of narrative feedback. The BENGI design also allows for quick exchanging and cleaning of components that encounter air from the patient’s lungs. Further, our device accomplishes all these goals with a total unit cost of < 100 USD.

The BENGI was successfully assembled utilizing inexpensive, off-the-shelf electronic components and 3D printed parts for the housing. The two AAAs and 5 V regulator were initially thought to be adequate power for the BENGI, yet initial testing demonstrated insufficient power when both the MP3 player and LED ring were powered simultaneously. For this reason, an additional external AAA battery pack was added. Future iterations of the BENGI design anticipate the use of a rechargeable lithium-ion battery, along with merging of electronic components to a single printed circuit board to allow for a more compact form factor, removing the need for an external battery pack and the mask connector, reducing instrument dead space.

Various tests were conducted to validate the flow and tidal volume measurements performed by the BENGI prototype. Bland–Altman analysis of the flow measurements demonstrated a bias for the BENGI to overestimate compared to the computer sensor by 4.4%. However, there is a clear positive trend present, indicating that the calculated flow bias is an underestimate of the true bias at higher flow rates and an overestimate at lower flow rates [34, 35]. This flow bias then carried over into the tidal volume calculations performed by the BENGI, and for all three inspiratory times tested, there were clear positive trends in the Bland–Altman plots as tidal volume increased. Furthermore, a decrease in inspiratory time from 1.0 to 0.5 s elevated the calculated bias from 2.1 to 3.9%, and an increase 1.0 to 2.0 s reduced the bias to 0.6%. Nevertheless, the greatest measured difference in calculated tidal volumes between the BENGI and computer were within 6% (target *V*_*T*_ = 750 mL at *t*_*insp*_ = 0.5 s), and this error was deemed acceptable, as it is well below the average healthcare provider’s error when delivering manual ventilation [20].

Additionally, an increase in average measured flow was correlated with a rise in tidal volume discrepancy between the BENGI and computer-measured tidal volumes. We thought that this discrepancy could be due to either a difference in (1) flow measurements and/or (2) sampling rates between the computer and BENGI flow sensors. The measured sampling rate of the BENGI was 713 Hz, with all samples above 700 Hz. Our simple computational simulation of varying sampling rate predicted a log–log linear correlation between tidal volume difference and sampling rate, where an increase in sampling rate leads to a reduction in tidal volume difference. The sampling rate is limited mostly by the capability of the microcontroller, and partly by the BENGI’s software implementation. The tidal volume difference at the BENGI’s sampling rate was predicted to be 7 μL, suggesting that sampling rate was not the main cause for the observed differences in tidal volume measurements between the computer and BENGI. However, when a constant flow was administered, there were small but appreciable differences between flow measurements with the BENGI and computer sensor that increased with higher flow rates. This difference in measurements persisted even when the BENGI and computer flow sensors were switched (data not shown), indicating that the difference is not inherent to the calibration of the flow sensors themselves. Thus, the discrepancy in tidal volume measurements between the BENGI and computer is likely a flow-dependent and not a sampling rate-dependent phenomenon.

The “*Bag slower*” audio was triggered at values slightly faster (approximately 100 ms) than the target trigger of 0.5 s at 500- and 300-mL tidal volumes. These bounds could not be tested at 750 mL since the flow rates required to achieve these fast inspiratory times in our lung model necessitated inspiratory pressures above the mechanical ventilator’s allowable pressure limit of 100 cm H_2_O. The “*Bag faster*” audio cue was also triggered at slightly faster (60 ms) than the target 2.0 s at 750 mL, within 60 ms faster or 30 ms slower at 500 mL, and within 160 ms faster or 30 ms slower at 300 mL. Further resolution to more precise upper and lower triggering time boundaries could not be achieved, as we were limited by the discrete inspiratory times that the mechanical ventilator provided for a given tidal volume. Similarly, the “*Leak detected*” signal lies between 43 to 50% leak at all three tested tidal volumes, slightly lower than the target leak trigger of 50%. Further resolution to these upper and lower trigger time bounds could hypothetically be achieved with a different mechanical ventilator that allows for more precise inspiratory times and higher inspiratory pressures (for the “*Bag slower*” and “*Bag faster*” triggers) or by drilling with a smaller drill bit (for the “*Leak detected*” trigger). However, the upper and lower bounds demonstrated may arguably represent satisfactory agreement with their respective targets, and any additional refinement would represent a clinically irrelevant improvement.

In the BENGI platform, several potential sources of error (artifacts from low sampling rate, system leaks) could be ruled out. However, differences in calibration between the computer flow sensor and the BENGI’s sensor remain a potential minor source of error. The sensors used are calibrated by the manufacturer for mixtures of air and oxygen, with alterations in humidity and temperature causing the most output variation [36].

Hard-coded values used in the BENGI’s software and testing parameters follow published recommendations from the American Heart Association (AHA), the European Resuscitation Council (ERC), and the International Liaison Committee on Resuscitation (ILCOR), which recommend adult tidal volumes of 500–600 mL delivered over 1 s at a rate of 10 breaths per minute [37, 38]. Using these parameters, several operational functions (audio cues for inspiratory speed and leak detection) have now been shown to operate as designed. Optimization of several parameters related to these additional functions is not ‘fixed’. For example, we set the optimal inspiratory time window to 0.5–2 s; however, other values of upper and lower limits may be appropriate under different sets of conditions, as extreme changes in tidal volume and respiratory rate requirements may require changes to flow and inspiratory time. In neonatal resuscitation, for example, inspiratory times may need to be much shorter to accommodate the necessary increase in respiratory rate. Similarly, the threshold for leak detection might be more beneficial at a lower percentage of the delivered tidal volumes. We selected the parameters used to show proof-of-concept; these may be altered after clinical testing.

One recognized limitation of the BENGI’s current design is the moderate amount of dead space that is introduced into the system (up to 20 mL), which becomes especially important in neonatal resuscitation. In adult patients with tidal volumes averaging 500 mL, 20 mL of dead space represents only 4% of tidal volume and can be considered relatively benign. For neonatal patients, this added dead space may represent 100% or more of the tidal volume, and thus would not be safe. Reduction in the size of the BENGI housing could eliminate the need for the extra tube currently used to attach the mask to the BENGI; this could reduce the dead space. This could be achieved by using custom printed circuit boards with a pre-programmed microcontroller, allowing for the integration of the current standalone electronic components. Another possible limitation is the significant power usage from the combination of LED ring and MP3 module. Later design iterations are in development which substitute the current MP3 module with an alternative that is optimized for low power consumption.

There were also limitations to the tidal volume tests; namely, the mechanical ventilator is programmed to prevent the delivery of inspiratory flow rates that result in inspiratory pressures exceeding 100 cm H_2_O, which prevented the testing of BENGI tidal volume accuracy above 750 mL at 0.5 s. This also prevented testing the “*Bag slower*” trigger at 750 mL. The large pressure gradient is likely a product of the significant flow rate required to deliver 750 mL in 0.5 s (1.5 L/s, or 90 L/min), as the only appreciable resistance in the system is the test lung, which mimics healthy human lung resistance. Resistance added to the circuit by the device is negligible compared to the resistance of the test lung and did not create significantly high peak pressures at lower flow rates. Practically, 90 L/min is far above a normal resting inspiratory flow rate (20 – 30 L/min), and delivery of 750 mL tidal volumes at such a speed would very rarely, if ever, be indicated.

Additionally, the tidal volumes tested (between 300 to 900 mL in 50 mL increments) would be appropriate in adult and some pediatric patients but not neonatal patients, where appropriate bagging may be an even more prevalent problem given the rapid changes in respiratory compliance immediately after birth, small tidal volumes – and therefore amplified problems with leaks – and high respiratory rates [39–41]. Further testing at tidal volumes more applicable to this group could demonstrate potential utility in these patients.

Other tidal volume feedback monitoring has recently been developed. You et al. likely developed the first of such devices in 2017, which measures airflow via displacement of a compression spring located within the device’s air channel, calculating and displaying delivered tidal volumes for each breath. While shown to be effective and accurate in a manikin study, this device does not allow for retrograde flow, necessitating an additional outflow valve [42]. MEDICON’s Amflow^®^ also uses a turbine and infrared sensor to measure airflow. The device’s display shows a countdown timer to guide respiratory rate, as well as tidal volume in the form of a bar whose size is dependent on the proportion of a set target volume [43]. Turbine-based airflow measurements can be relatively inaccurate at both high and low flows, where turbine inertia can introduce measurement error. The Real-Time Ventilation Feedback Device (RTVFD) by Heo et al. utilizes a mass flow sensor to measure flow rate and calculate the delivered tidal volume, which is numerically displayed on an onboard screen alongside a breath countdown timer [44]. Finally, the Ventilation Feedback Device (VFD) developed by Khoury et al. gives visual tidal volume and respiratory rate feedback, along with visual information on inspiratory/expiratory times and percent air leakage [45].

Although these tidal volume feedback devices can reduce manual hyperventilation, they can also distract healthcare personnel, which can be especially detrimental during resuscitation [46]. While detailed data on several parts of a patient’s respiratory physiology may be useful in certain situations, interpreting more complex numerical and textual visual data during a high-stress emergent situation can be distracting and overwhelming. Thus, the BENGI’s extremely simplified visual LED and spoken audio cues may be more rapidly and easily interpretable, with a reduction in mental workload. Further study is needed to quantify and compare the mental workload placed on healthcare personnel while using the BENGI versus other similar devices during simulated CPR scenarios.

Novel features of the BENGI in comparison to existing ventilation feedback devices include its use of low complexity components and narrative audio feedback. To our knowledge, there are no other devices providing narrative audio feedback to guide manual ventilation technique. Here, we have shown that the BENGI accurately measures and report tidal volumes and properly trigger its audio cues. However, we have not demonstrated that the BENGI can improve bagging efficacy and safety by the end user (i.e., healthcare personnel), which is another limitation of the current study. Future work includes a manikin study to validate the BENGI’s efficacy in improving healthcare providers’ bagging technique. This is a critical next step to demonstrate the potential utility of the BENGI in a clinical setting. Additionally, as a medical device, the BENGI will eventually require regulatory approval, and further work would require modification of design and clinical testing to meet appropriate FDA and other regulatory agencies standards.

## Conclusion

The BENGI is a handheld tidal volume monitoring device constructed with low-cost components intended for use with BVM systems in emergent manual ventilation. Ease of use, accessibility, and low cost were main motivating factors in design decisions and set it apart from existing tidal volume monitoring devices, along with its narrative feedback. Testing has demonstrated that the BENGI accomplishes the core functions of existing tidal volume monitors with comparable accuracy while maintaining a low cost and maximizing ease of use. By providing real-time, objective feedback to healthcare providers performing manual ventilation, the BENGI has the potential to significantly reduce sequelae associated with hyperventilation.

## Supplementary Information

**Additional file 1.**

## Data Availability

Complete datasets, detailed design documents, and code may be requested from the corresponding author.
