# Supplementary material for: Experimental validation of a portable tidal volume indicator for bag valve mask ventilation
Source: BMC Biomed Eng. 2022 Nov 17;4:9. doi: 10.1186/s42490-022-00066-y (PMC9668705; doi:10.1186/s42490-022-00066-y)
Supplement: Supplementary file 1 — Additional file 1. [file 42490_2022_66_MOESM1_ESM.pptx]

## Slide 1
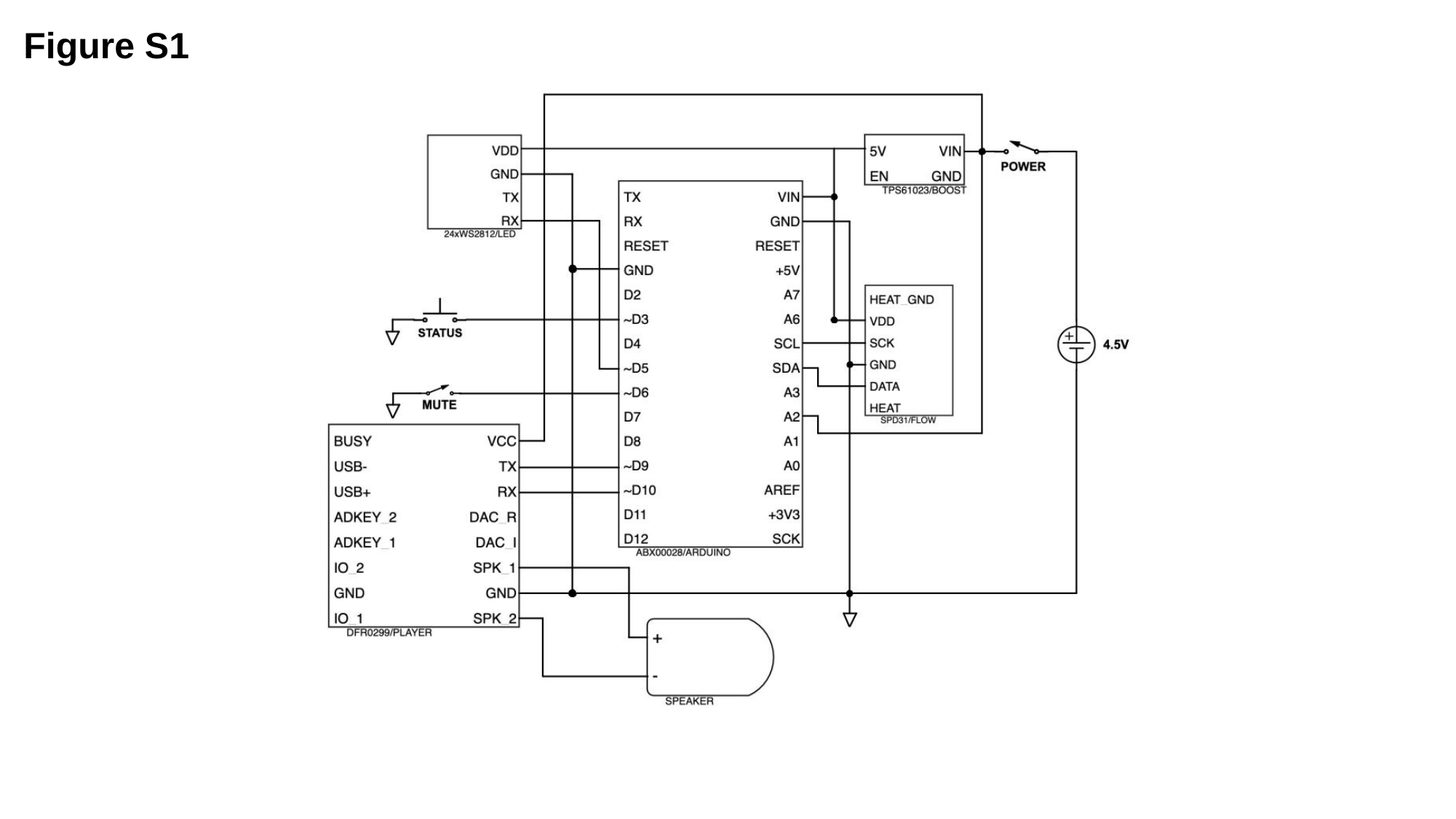

Figure S1

## Slide 2
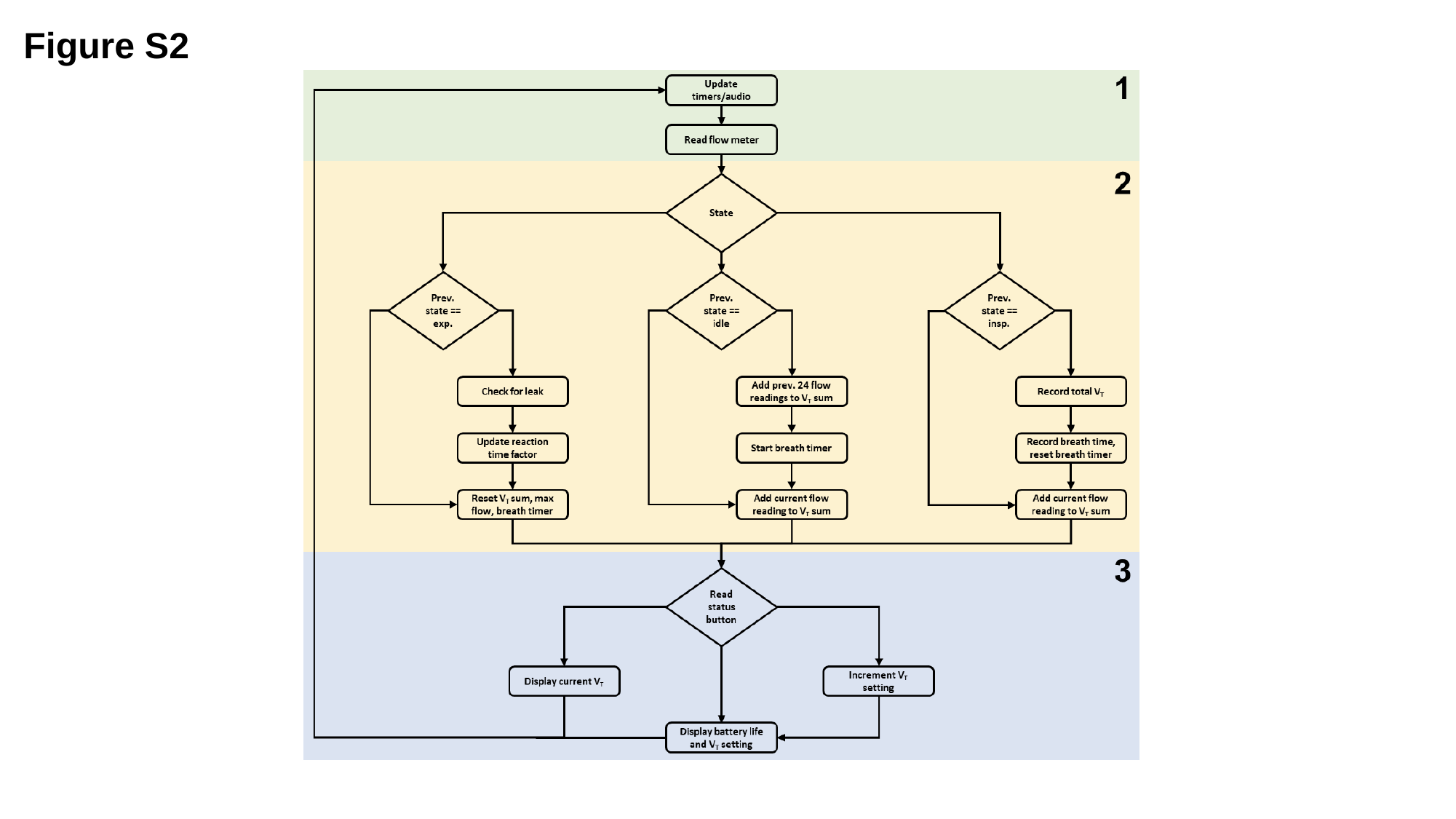

Figure S2

## Slide 3
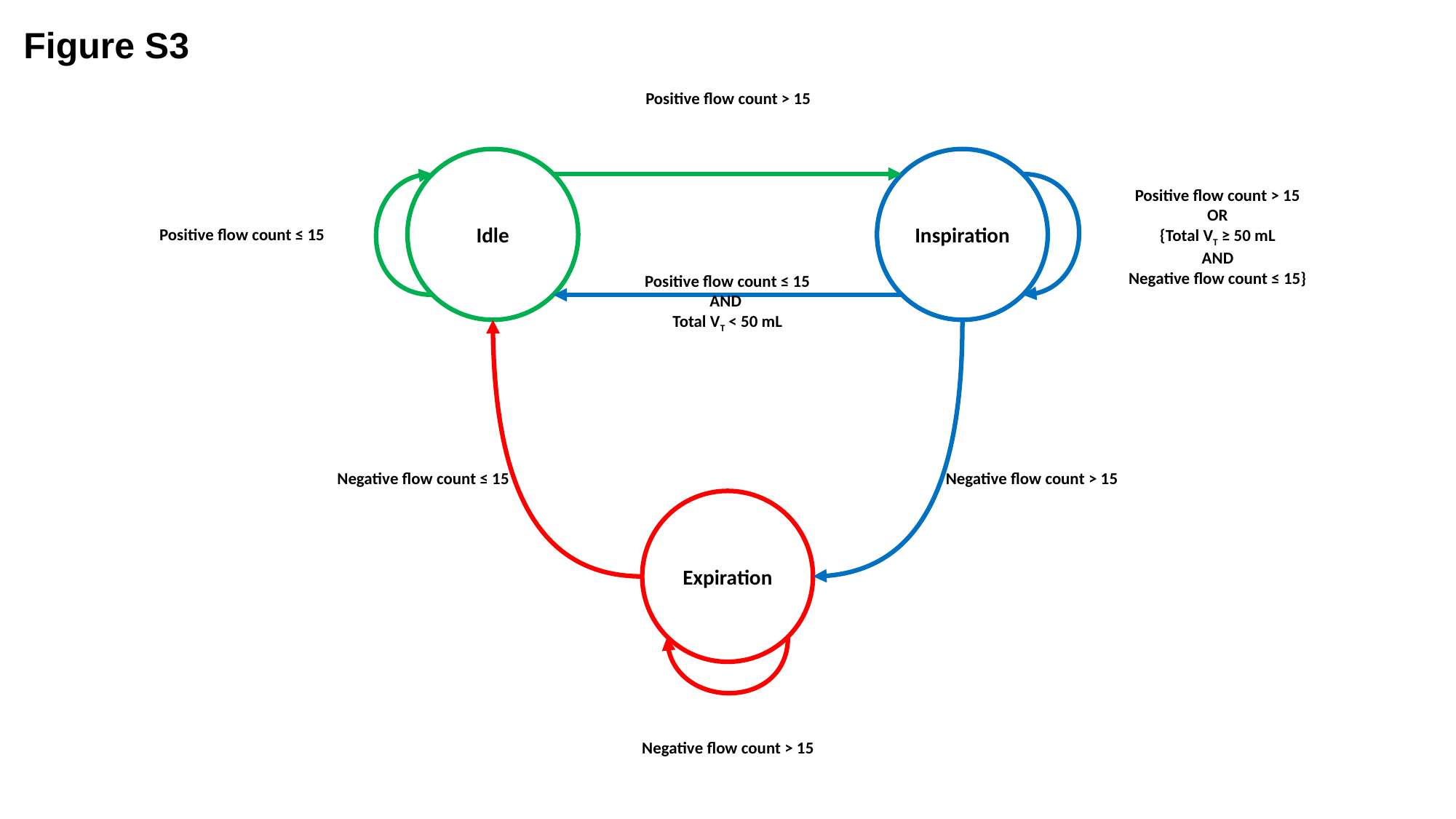

Figure S3
Positive flow count > 15
Idle
Inspiration
Positive flow count > 15
OR
{Total VT ≥ 50 mL
AND
Negative flow count ≤ 15}
Positive flow count ≤ 15
Positive flow count ≤ 15
AND
Total VT < 50 mL
Negative flow count > 15
Negative flow count ≤ 15
Expiration
Negative flow count > 15
